# Contributions of differential p53 expression in the spontaneous immortalization of a chicken embryo fibroblast cell line

**DOI:** 10.1186/1471-2121-7-27

**Published:** 2006-06-30

**Authors:** Shelly A Christman, Byung-Whi Kong, Megan M Landry, Hyunggee Kim, Douglas N Foster

**Affiliations:** 1 Department of Animal Science, University of Minnesota, St. Paul, MN 55108, USA; 2 Division of Biosciences and Technology, Korea University, Seoul, 136–701, Korea

## Abstract

**Background:**

The present study was carried out to determine whether the p53 pathway played a role in the spontaneous immortalization of the SC-2 chicken embryo fibroblast (CEF) cell line that has been in continuous culture for over three years.

**Results:**

The SC-2 cell line emerged from an extended crisis period with a considerably slower growth rate than primary CEF cells. The phenotype of the SC-2 cells changed dramatically at about passage 80, appearing smaller than at earlier passages (e.g., passage 43) and possessing a small, compact morphology. This morphological change coincided with an increase in growth rate. Passage 43 SC-2 cells expressed undetectable levels of p53 mRNA, but by passage 95, the levels were elevated compared to primary passage 6 CEF cells and similar to levels in senescent CEF cells. However, the high level of p53 mRNA detected in passage 95 SC-2 cells did not correlate to functional protein activity. The expression levels of the p53-regulated p21^WAF1 ^gene were significantly decreased in all SC-2 passages that were analyzed. Examination of the Rb pathway revealed that E2F-1 and p15^INK4b ^expression fluctuated with increasing passages, with levels higher in passage 95 SC-2 cells compared to primary passage 6 CEF cells.

**Conclusion:**

The present study suggests that altered expression of genes involved in the p53 and Rb pathways, specifically, p53 and p21^WAF1^, may have contributed to the immortalization of the SC-2 CEF cell line.

## Background

Normal cells cultured *in vitro *undergo a characteristic number of divisions before entering a non-dividing state termed senescence [1]. Most cells are unable to overcome senescence to continue dividing unless key tumor suppressor pathways are first altered. Cellular immortalization has been achieved by the inactivation of the p53 and Rb pathways or by the activation of telomerase [2,3]. While spontaneous immortalization is an extremely rare event in human cells [4], rodent cells spontaneously immortalize at much greater rates [2]. For avian cells, spontaneous immortalization also has been very rare, with only two other spontaneously immortalized chicken cell lines reported (DF-1 [5-8] and SC-1 [9]).

The tumor suppressor gene, p53, has often been referred to as the 'cellular gatekeeper' or the 'guardian of the genome' because of its role in cell cycle arrest and apoptosis in response to cellular damage [10,11]. The p53 protein is dysfunctional in most human cancers, with p53 itself being mutated in about half of these cancers. In the other half of cancers when p53 itself is not mutated, it is inactivated indirectly as a result of alterations in the gene products that interact with p53 or transmit information to or from p53 [12]. In normal cells, the main negative regulator of p53, MDM2, maintains a relatively short p53 half-life by targeting it for degradation, thus preventing it from exerting antiproliferative effects [13]. The MDM2 and p53 proteins operate in a feedback loop whereby MDM2 degrades p53 while p53 activates transcription of MDM2, thereby helping to maintain low p53 levels in normal cells [14]. Another important gene involved in the p53 pathway is ARF, which binds directly to MDM2, protecting p53 from degradation and allowing for the increased expression of p53 [15,16]. One study showed that senescence requires an increase in the levels of ARF and that conversely, fibroblasts lacking ARF proliferate indefinitely [17]. Many signals activate p53 such as DNA damage, telomere shortening, hypoxia, and aberrant proliferative signals. Upon activation, p53 induces a G1/S phase cell cycle arrest that is mediated by p21^WAF1 ^[18,19], which functions by inhibiting the CDKs (cyclin-dependent kinases) during the G1 phase of the cell cycle, thereby preventing cell cycle progression [20-22]. Consistent with this, the levels of p21^WAF1 ^have been reported to be elevated in senescent cells [23].

The other main tumor suppressor mechanism involves the Rb pathway, and alterations in this pathway have been shown to lead to tumorigenesis [24]. Throughout the cell cycle Rb undergoes changes in phosphorylation, with a hyperphosphorylated form predominant in proliferating cells and a hypophosphorylated form predominant in quiescent or differentiating cells [25]. As Rb is hyperphosphorylated by CDK-cyclins in the mid-to-late G1 phase of the cell cycle, Rb is unable to bind and inhibit the actions of E2F-1, allowing E2F-1 to promote cell cycle progression from the G1 phase of the cell cycle into the S phase [26]. Senescent cells express high levels of the CDK-inhibitor, p16^INK4a^, which prevents CDK-cyclins from phosphorylating Rb, keeping E2F-1 bound to Rb and unable to promote entry into the S phase of the cell cycle [27,28]. The p16^INK4a ^gene is inactivated in many tumor cell lines [29-31], while overexpression of p16^INK4a ^results in G1 arrest [32].

In the present study, mRNA and protein expression levels of various genes involved in the p53 and Rb pathways were measured in multiple passages of a spontaneously immortalized CEF cell line, SC-2. By analyzing the expression of important cell cycle regulatory genes at various stages of the immortalization process it was observed that the SC-2 cells underwent an initial crisis period between passages 18 and 33 and another transition period at about passage 80. From this data, it has been hypothesized that following the initial crisis period, changes in the expression of important cell cycle regulatory genes allowed the cells to continue growing. It is quite possible that additional coordinated fluctuations in gene expression allowed the cells to increase their growth rate at approximately passage 80 and to become fully immortal.

## Results and discussion

### Growth rate and morphological analysis

In the present study the third reverse transcriptase (RT)-negative spontaneously immortalized chicken cell line (SC-2) was established. Data were obtained from many different passages of SC-2 cells, but only shown for passages 43 and 95, since they represent the major transition phases that occurred in the SC-2 cells. An examination of this type has been performed for another spontaneously immortalized cell line (SC-1) [9], but the expression of most cell cycle regulatory genes were very different compared to the SC-2 cells.

**Figure 1:**
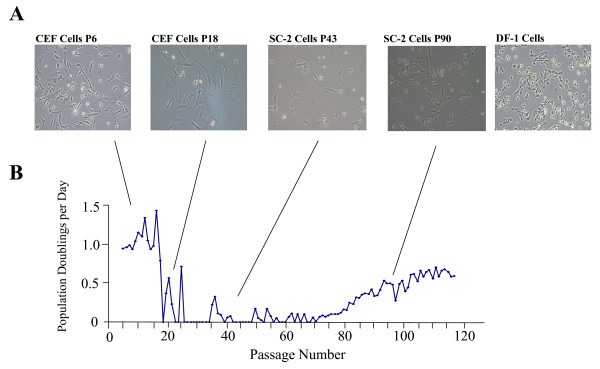
**Morphology and growth curve of primary and immortal CEF cells. **A) Representative photomicrographs of primary passage 6 CEF cells, senescent passage 18 CEF cells, passage 43 SC-2 cells, passage 95 SC-2 cells and immortal passage 280 DF-1 cells **. B) **Growth curve of SC-2 cells. SC-2 cells were plated at a density of 3 × 10^5 ^cells/10 cm dish. At 80% confluency cells were trypsinized, counted and the number of population doublings per day calculated.

The SC-2 cell line was derived from primary CEF cells that were thawed at passage 4. Between passages 5 and 17 the growth rate of the CEF cells averaged 1.03 population doublings per day (pd/d). The cells entered a crisis period between passages 18 and 33, during which their growth rate was negligible except for measurable growth between passages 20 and 21. During this crisis period, cells were refed every 2–3 days and passaged until small foci of cells appeared. At about passage 33 a subpopulation of cells emerged that was able to overcome cellular senescence and continue growing when other cells in the population succumbed to cell death. These cells were considered to have an extended lifespan and as such were designated the SC-2 cell line. The SC-2 cells continued to grow slowly between passages 33 and 80 averaging 0.12 pd/d. Passage 43 SC-2 cells were flattened and enlarged compared to the presenescent CEF cells. The phenotype of the SC-2 cells changed dramatically at about passage 80, appearing smaller than observed earlier at passage 43 and possessing a small, compact morphology, similar to DF-1 cells (Fig. 1A). Coinciding with the dramatic morphological change at passage 80, the growth rate of the SC-2 cells increased to an average of 0.60 pd/d after passage 80 (Fig. 1B). The SC-2 cells are currently at passage 120 and have maintained the improved morphology and increased growth rate since passage 80. The SC-2 cells displayed contact inhibition when grown to confluence, did not show evidence of multi-layered growth, and did not form colonies when grown in a semi-solid agarose suspension (data not shown), suggesting that these cells lacked many of the properties of transformed cells. Karyotypic analysis (data not shown) of the SC-2 cells indicated that they displayed a normal diploid karyotype. The SC-2 cells were analyzed twice for RT activity and mycoplasma contamination and found to be negative for both (data not shown).

**Figure 2:**
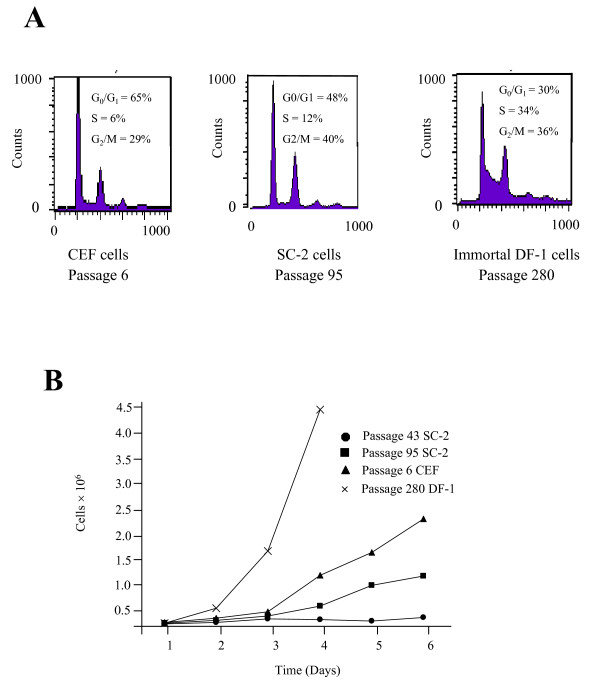
**FACS analysis and growth rates of primary and immortal CEF cells**. A) Cell cycle analysis of pre-senescent passage 6 CEF, passage 95 SC-2, and immortal passage 280 DF-1 cells was performed using fluorescence-activated cell sorting (FACS) of propidium iodide-stained cells. **B) **To determine growth rates, primary CEF (passage 6) and immortal CEF (passage 43 SC-2, passage 95 SC-2, and passage 280 DF-1) cells were plated at a density of 1 × 10^5 ^cells/10 cm dish using conditions as described in Materials and Methods. Both primary and immortal CEF cells were counted each day for up to 6 days to determine cell numbers. The immortal DF-1 cells are only shown through day 4 due to their relatively higher cell numbers.

FACS analysis showed that SC-2 cells possessed a distinct cell cycle distribution compared to either primary passage 6 CEF or immortal DF-1 cells (Fig. 2A). The percentage of cells in the G1 phase of the cell cycle was 65% for primary passage 6 CEF cells, 48% for SC-2 cells, and 30% for immortal passage 280 DF-1 cells.

Cell growth rates were compared as shown in Figure 2B. Passage 43 SC-2 cells had a very slow growth rate, with only 0.17 pd/d after six days, while passage 95 SC-2 cells showed increased growth rates (0.60 pd/d) that where more similar to primary CEF cells (0.72 pd/d). The DF-1 cells displayed the highest growth rate at 1.33 pd/d after four days.

### Analysis of p53 and p53-regulated genes

**Figure 3:**
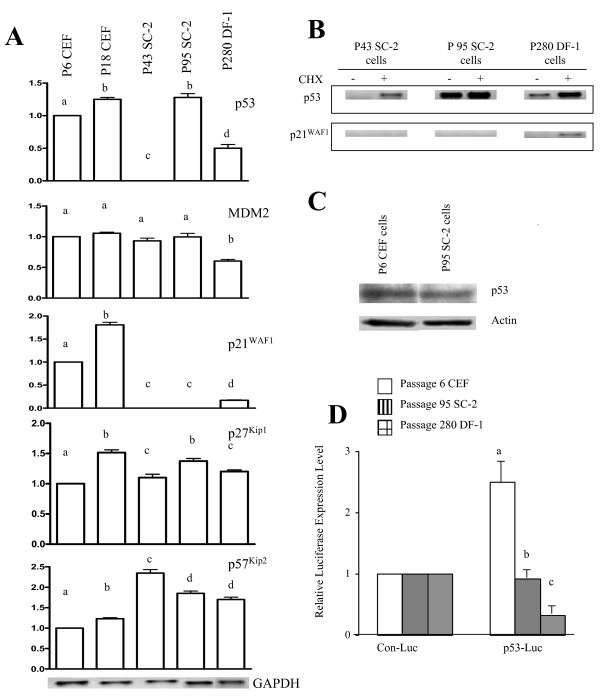
Analysis of the p53 pathway using RT-PCR and a p53-Luciferase reporter assay.A) Relative levels of mRNA expression for genes involved in the p53 pathway determined by RT-PCR. For each gene the expression level of passage 6 CEF cells was set to 1.0. The levels for other samples were then adjusted accordingly. A minimum of three independent RT-PCR experiments were analyzed using the NIH image software program and normalized using the expression levels of GAPDH. Differences ≤0.05 were considered significant. Different letters indicate significant differences. B) p53 and p21^ WAF1 ^mRNA expression levels were measured by RT-PCR in passage 43 and 95 SC-2 cells as well as in passage 280 DF-1 cells ± the protein synthesis inhibitor CHX. C) p53 protein levels in primary passage 6 CEF and passage 95 SC-2. A minimum of three independent Western blot experiments were analyzed using the NIH image software program and normalized using the expression levels of actin. Differences ≤ 0.05 were considered significant. D) Altered biological activity of p53 in immortal DF-1 and SC-2 cells using the luciferase reporter gene assay. All cells were cotransfected with a control reporter plasmid (Con-Luc) or p53 reporter plasmid (p53-Luc) and pcDNA3.1-LacZ plasmid. The β-lactamase (LacZ) gene was amplified by PCR to normalize transfection efficiency and the GAPDH gene was amplified by RT-PCR to account for loading differences. Levels of luciferase activity were determined using the Luciferase Assay System (Promega) following the manufacturer's instructions. Luciferase activity in the cells transfected with the Con-Luc plasmid were set to 1.0. The normalized values shown are relative expression levels with s.d. Differences ≤ 0.05 were considered significant. Different letters indicate significant differences.

Because of the importance the p53 pathway plays in controlling the cell cycle, an examination of p53 and its related genes was performed during the conversion process in the SC-2 cell line using RT-PCR and Southern blot analysis. Like many other studies, an increase in p53 mRNA expression (25%) was observed in normal senescent passage 18 CEF cells compared to primary passage 6 CEF cells (Fig. 3A). The decreased p53 mRNA expression in passage 43 SC-2 cells agrees with other studies that have observed decreased expression of p53 in immortal cells [8,33]. Unexpectedly, increased p53 levels were observed in passage 95 SC-2 cells that were similar to levels in senescent passage 18 CEF cells. One possible explanation for this observation is the functional inactivation of p53 via deletion or point mutations. However, the nucleotide sequence of p53 mRNA in the SC-2 cells was identical to that of the published chicken p53 mRNA sequence (GenBank: X13057) for the first portion of the 5'-end and the remainder of the 3'-end. We have had difficulties in sequencing the remaining approximate 250 bp (between nucleotides 242 and 494), which is predominantly double-stranded [34]. With over 80% of the molecule sequenced, it appears that there are no deletions or mutations that exist. These results suggest that other cellular modifications may be responsible for the altered activity of p53 in the SC-2 cells.

To determine whether the fluctuation in p53 mRNA levels affected other genes involved in the pathway, mRNA expression levels of MDM2, which is the predominant negative regulator of p53, were examined. Although the MDM2 gene is upregulated in 30%-40% of human tumors [35], the levels of MDM2 in the SC-2 cells were similar to levels observed in normal primary passage 6 CEF cells. The levels of MDM2 were expected to be higher in passage 95 SC-2 cells since p53 mRNA levels were elevated. A possible explanation for the unchanged levels of MDM2 in the present study was that p53 protein was inactive and not able to participate with MDM2 in the normal feedback loop.

Another important target of p53 is p21^WAF1^, a gene that causes arrest at the G1 and G2 phases of the cell cycle. In order to assess whether or not the fluctuating p53 levels were functional, mRNA expression of p21^WAF1 ^was measured. Similar to other studies [36,37], levels of p21^WAF1 ^dramatically increased in senescent passage 18 CEF cells (82%; Fig. 3A). Significant down-regulation of p21^WAF1 ^mRNA was observed in all passages of SC-2 cells compared to primary passage 6 CEF cells. Since the p21^WAF1 ^gene is up-regulated by p53 in response to DNA damage [19], the fact that the SC-2 cells possessed extremely low expression levels of p21^WAF1^, even in the presence of elevated levels of p53 mRNA, suggested that p53 may be functionally inactive.

The related gene p27^Kip1^, which belongs to the same family as p21^WAF1 ^(the Kip/Cip family), is a CDK inhibitor that prevents transition from the G1 to S phase of the cell cycle. Similar to p21^WAF1^, levels of p27^Kip1 ^also increased at senescence in normal CEF cells (45%; Fig. 3A). The levels of p27^Kip1 ^in the immortal DF-1 cells and the SC-2 cells at passage 43 were slightly elevated. However, by passage 95, levels of p27^Kip1 ^in the SC-2 cells had increased by almost 40% over levels observed in primary passage 6 CEF cells.

Another member of the Kip/Cip family is p57^Kip2^, which when over-expressed, has been shown to lead to growth arrest at the G1 phase of the cell cycle in two different cell lines [38,39]. Conversion of a lifespan extended population of human mammary epithelial cells to a fully immortal phenotype was marked by a dramatic decrease in p57^Kip2 ^expression, indicating that p57^Kip2 ^may be used as a marker of fully immortal cell lines [40]. Here p57^Kip2 ^levels were slightly increased (11%) in senescent passage 18 CEF cells (Fig. 3A). However, in contrast to others who have observed decreased expression of p57^Kip2 ^mRNA in immortal cell lines [41], the SC-2 cells at passages 43 and 95 and the immortal DF-1 cells possessed significantly increased levels of p57^Kip2^. While the levels of p57^Kip2 ^in passage 43 SC-2 cells were increased by 134% over primary passage 6 CEF cells, the passage 95 SC-2 cells still possessed levels of p57^Kip2 ^that were 85% greater than the levels expressed in primary passage 6 CEF cells, but these levels had decreased considerably from the levels measured in passage 43 SC-2 cells. This suggested that the SC-2 cells still may be in the process of converting to a fully immortal phenotype.

To determine if the altered p53 transcriptional activity in SC-2 cells was caused by an inhibitory/repressor protein, cells were treated with the protein synthesis inhibitor, CHX, which has been shown to stabilize several mRNAs [42]. It was previously shown that CHX treatment restored p53 mRNA levels in CEF cells and in immortalized (10)3 and (10)7 murine embryonic fibroblast (MEF) cells expressing constitutively low levels of p53 mRNA [8]. We reasoned that a translation inhibitor such as CHX should block the function of translation-dependent p53 regulatory elements that may be present, resulting in increased p53 mRNA levels. Since primary passage 6 CEF cells possessed normal levels of p53 mRNA it was not surprising that CHX had no effect on p53 expression (data not shown). However, in cells that contained depressed levels of p53 (passage 43 SC-2 and DF-1 cells), CHX treatment resulted in an upregulation of p53 mRNA expression (Fig. 3B). This suggested that both the passage 43 SC-2 cells and DF-1 cells contained a p53-regulatory element that blocked expression of p53 resulting in a 5- and 2.5-fold increase in mRNA expression following CHX treatment, respectively. Removal of this putative p53 inhibitor via CHX allowed expression levels of p53 mRNA to increase. However, CHX had no effect in passage 95 SC-2 cells, which already possessed elevated levels of p53 mRNA compared to primary passage 6 CEF cells. An examination of the effect of CHX on the expression of p21^WAF1 ^would help substantiate the functional status of p53 in the passage 95 SC-2 cells. However, treatment with CHX had no effect on the expression of p21^WAF1 ^mRNA expression in SC-2 cells (Fig. 3B). This indicates that the p53 mRNA levels in passage 95 SC-2 cells and the CHX-restored levels in passage 43 SC-2 cells were functionally inactive.

Thus far, indirect assessment of the functional activity of p53 has been demonstrated by measurement of the expression of p53-regulated genes. Since the ability of p53 to activate or repress transcription of downstream genes is important for cell cycle control, the protein level of p53 and the direct functional activity also were measured. Unlike the mRNA data that showed an increase in p53 mRNA expression in passage 95 SC-2 cells, Western blot analysis showed a slightly decreased level of p53 protein in passage 95 SC-2 cells (Fig. 3C). To determine whether the protein levels of p53 in the passage 95 SC-2 cells were functional, direct activity was measured after transfection of a p53 consensus binding sequence promoter/luciferase reporter plasmid construct. It was discovered that the functional protein activity of p53 was 2.8-fold less than that of primary passage 6 CEF cells (Fig. 3C). The functional inactivation of p53 protein in the passage 95 SC-2 cells likely allowed the cells to escape the growth constraints imposed by p53. In the DF-1 cells the functional p53 activity was greatly decreased compared to the level in primary passage 6 CEF cells, which is in agreement with the lower mRNA expression levels observed in the DF-1 cells.

### Expression analysis of genes involved in the Rb pathway

**Figure 4:**
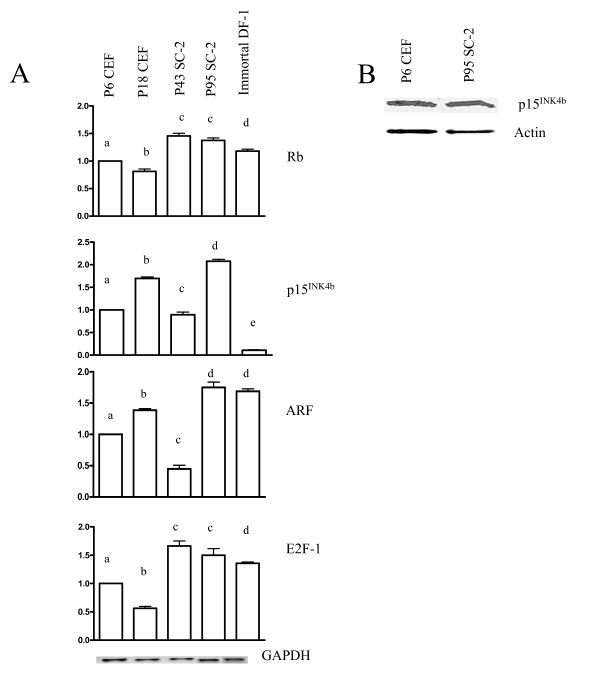
**Analysis of the Rb pathway**. A) Levels of mRNA expression for genes involved in the Rb/p16^INK4a ^pathway measured by RT-PCR. For each gene, the expression level was set to 1.0 for passage 6 CEF cells. The levels for other samples were then adjusted accordingly. A minimum of three independent RT-PCR experiments were analyzed using the NIH image software program and normalized using the expression levels of GAPDH. Differences ≤ 0.05 were considered significant. Different letters indicate significant differences. **B) **p15^INK4b ^protein levels in primary passage 6 CEF, and passage 95 SC-2 cells. A minimum of three independent Western blot experiments were analyzed using the NIH image software program and normalized using the expression levels of actin. Differences ≤ 0.05 were considered significant.

Another tumor suppressor that plays a fundamental role in controlling cell cycle progression and senescence is Rb. Alterations in this pathway are second only to alterations in the p53 pathway in human cancers [43]. Surprisingly, an up-regulation of Rb expression (~40%) was observed in the SC-2 cells compared to primary passage 6 CEF cells (Fig. 4A). However, this is in agreement with the results of a previous study that showed up-regulated levels of Rb mRNA in the immortal DF-1 cell line [44]. The up-regulation of Rb expression that was observed in all passages of SC-2 cells examined may be the result of a functionally inactive p53, as others have shown that Rb mRNA expression is repressed by p53 in mammalian cells [45].

The tumor suppressor p16^INK4a^, a key regulator of Rb that maintains Rb in a hypophosphorylated state, is inactivated in many transformed cell lines [29-31]. Since p15^INK4b ^assumes the role of p16^INK4a ^in chickens [46], its mRNA levels were measured. Unexpectedly, only a slight decrease in p15^INK4b ^expression in passage 43 SC-2 cells and a significant increase in passage 95 SC-2 cells were observed. The fact that the passage 95 SC-2 cells possessed an increased expression of p15^INK4b ^suggested that the Rb pathway may be altered in the SC-2 cell line. Hypermethylation of the p15^INK4b ^promoter in the SC-2 cells is unlikely since it would result in the downregulation of p15^INK4b ^mRNA expression, which was not observed in the SC-2 cells. It is possible that p15^INK4b ^had been inactivated in the SC-2 cells, unlike the DF-1 cells where hypermethylation of the p15^INK4b ^gene promoter causes the downregulation of p15^INK4b ^mRNA expression [59]. To determine whether the increased levels of p15^INK4b ^mRNA were translating normal protein levels, Western blot analysis was used to determine the levels of p15^INK4b ^protein in the SC-2 cells. As shown in Fig. 4B the p15^INK4b ^protein levels also were increased (30%) in the SC-2 cells compared to primary passage 6 CEF cells.

The p15^INK4b ^alternate reading frame sequence (ARF), is encoded by the same genetic locus as p15^INK4b^, but in a different reading frame, resulting in two completely different genes. The chicken ARF gene was recently isolated, and it was determined that chicken ARF binds to MDM2 and stabilizes p53, just as it does in human and mouse [59]. To verify the role of ARF in the immortalization process of chicken cells, levels were compared in primary and immortal CEF cells. As predicted, the levels of ARF were elevated in senescent passage 18 CEF cells and decreased in passage 43 SC-2 cells (Fig. 4A). Although increased expression of ARF has been shown to induce cell cycle arrest [47], this was not observed in the passage 95 SC-2 or DF-1 cells. It has been shown that E2F-1 (an important gene in the Rb pathway) directly activates the expression of ARF, establishing a link between the Rb and p53 pathways [48]. E2F-1 is one of the growth-regulatory genes that is suppressed in senescent human cells [49,50] and can induce senescence when over-expressed in normal human fibroblasts via ARF. This induced senescence is dependent upon the presence of a functional p53 gene [51]. In the present study, elevated levels of E2F-1 were observed in immortal SC-2 and DF-1 cells, which is similar to other immortal chicken cell lines developed in our lab [8]. The study by Bates et al. [48] proposed that induction of ARF by E2F-1 would stabilize p53 unless there existed a mutation in ARF or in p53 itself, again supporting our hypothesis that p53 was inactive in the SC-2 cells.

### Expression analysis of various cell cycle regulatory genes

**Figure 5:**
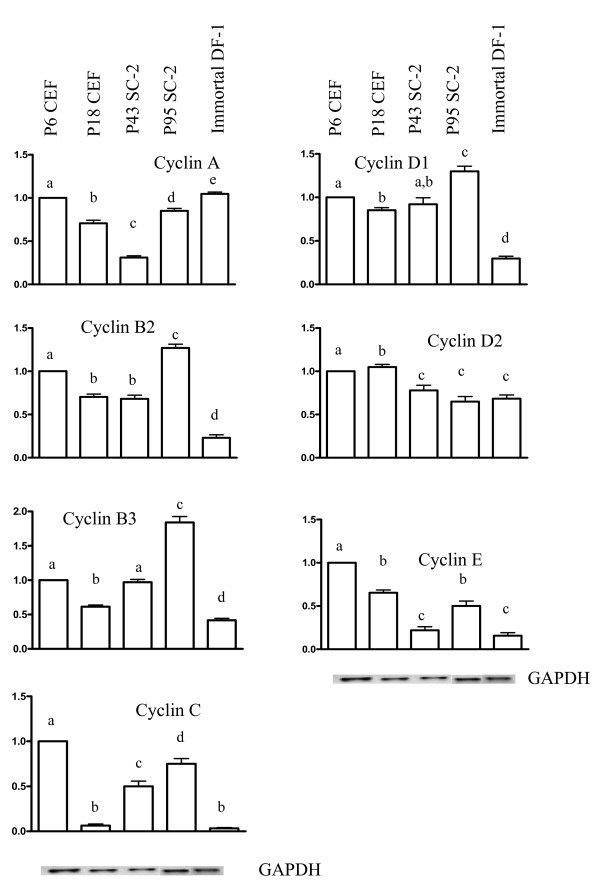
**Levels of mRNA expression for the various cyclins**. For each gene, the expression level was set to 1.0 for passage 6 CEF cells. The levels for other samples were then adjusted accordingly. A minimum of three independent RT-PCR experiments were analyzed using the NIH image software program and normalized using the expression levels of GAPDH. Differences ≤ 0.05 were considered significant. Different letters indicate significant differences.

Since the cell cycle also is regulated by the various cyclins [43], we examined mRNA expression levels of multiple chicken cyclin genes (Fig. 5). As expected, decreased expression for most of the cyclins was observed in senescent passage 18 CEF cells compared to primary passage 6 CEF cells. The only cyclin that was up-regulated was cyclin D2, which has been shown to increase its expression in growth-arrested cells [52]. In passage 43 SC-2 cells, the expression levels of most of the cyclins also were decreased, except for cyclin B3, which had similar levels compared to passage 6 CEF cells. However, by passage 95 the expression of cyclins B2, B3 and D1 were increased, while the expression of cyclins A, C, D2, and E were decreased compared to primary passage 6 CEF cells. Although unexpected, the decreased expression of certain cyclins in the SC-2 cells was also found in the fully immortal DF-1 cells (Fig. 5).

### Telomere regulation

Telomerase activation has been shown to be an important step in the immortalization of normal fibroblasts [53]. However, in the current study we were unable to show telomerase activation using the TRAP assay. Previous attempts to show telomerase activity in other immortal CEF cells [8,9], including the fully immortal DF-1 cell line (passage 280) have proven unsuccessful as well. Although telomerase activity was undetectable in the immortal SC-2 or DF-1 cells, telomere length did not decrease, even in senescent passage 18 CEF cells (data not shown). These results suggested that perhaps alterations in the p53 and/or Rb pathways may be more important than telomerase activation for the immortalization of CEF cells, which was in agreement with our other spontaneously immortalized cell lines (SC-1 and DF-1). The question still remains as to how chicken cells maintain telomere length without detectable telomerase activity. It is possible that sufficient telomerase activity is present in immortal CEF cells to maintain telomere length, but that this level is not high enough to be detected by the TRAP assay. It also is possible that telomere length is maintained in CEF cells through alternative mechanisms, as has been proposed in a number of human cells lines that do not display telomerase activity [54,55]. Although telomerase activity was undetectable, mRNA expression of TRF2, which is involved in the maintenance of telomeres, was elevated in passage 95 SC-2 cells (data not shown) suggesting that TRF2 may participate in the stabilization of telomere length in SC-2 cells.

## Conclusion

Unlike other studies that have introduced exogenous genes into cells in attempt to achieve immortalization or else have characterized a spontaneously immortalized cell line after it had been established, the spontaneous immortalization of the SC-2 cell line was analyzed as it transitioned from a life-span extended population of cells into a fully immortal cell line. Our results indicated that an initial down-regulation of p53 allowed the SC-2 cells to bypass senescence. The elevated levels of p53 mRNA in passage 95 SC-2 cells were shown to be partially non-functional using the luciferase reporter assay, and also verified by the significantly down-regulated p21^WAF1 ^expression that was observed. In fact, it has been shown that the loss of p21^WAF1 ^allowed cells to bypass senescence in spite of p16^INK4a ^expression [56], which is what was observed in the SC-2 cells. The inactivation of p53, and consequently p21^WAF1^, likely contributed to the SC-2 cells becoming a fully immortal cell line.

## Methods

### Cells and culture conditions

Primary and immortal CEF cells were grown in Dulbecco's Modified Eagle Medium (DMEM) high glucose enriched with 10% fetal calf serum (FCS), 1% penicillin-streptomycin, and 2 mM L-glutamine. All cell culture reagents were purchased from Invitrogen (Carlsbad, CA). The spontaneously immortalized DF-1 CEF cell line was derived from East Lansing Line 0 (*ev*-0) leghorn layer embryos [Avian Disease and Oncology Laboratory, East Lansing, MI) [5,6,7,8]. The spontaneously immortalized SC-2 CEF cell line was derived from SPAFAS eggs (specific pathogen free avian supply, Charles River Laboratories, North Franklin, CT). For the inhibition of translation studies, primary and immortal cells were incubated in 10% FCS-DMEM supplemented with 10 μg/ml of cycloheximide (CHX; Sigma, St. Louis, MO) for 4 h.

### Cell growth and cell cycle analysis

To determine growth rates, primary (passage 6 and 18) and immortal (DF-1 and SC-2) CEF cells were plated at a density of 3 × 10^5 ^cells/10 cm dish using culture conditions as described above. At 80% confluency, cells were trypsinized, counted, and the number of population doublings per day (pd/d) calculated. Cell growth also was determined by plating cells at a density of 1 × 10^5 ^cells/10 cm dish using conditions as described above, and counting cells each day for up to 6 days to determine cell numbers. Cell cycle analysis was carried out using FACS of propidium-iodide-stained cells and a Cell Quest software program (Becton Dickinson, Franklin Lakes, NJ) as described previously [57].

### Cell line characterization

Karyotypic analysis of cells was conducted by the Veterinary Diagnostic Laboratory at the University of Minnesota. The SC-2 cells were analyzed twice for reverse transcriptase (RT) activity using the EnzCheck RT Assay kit (Molecular Probes/Invitrogen Inc., Eugene, OR) according to the manufacturer's instructions. Mycoplasma contamination was analyzed using the Mycoplasma Plus™ kit (Stratagene, La Jolla, CA) according to the manufacturer's instructions.

### RT-PCR analysis

For semi-quantitative RT-PCR, 3 μg of DNase I-treated RNA was converted to cDNA using Superscript II reverse transcriptase (Invitrogen, Carlsbad, CA) following the manufacturer's instructions. A portion (1 μl) of the reverse transcription reaction was used to amplify cDNA fragments with chicken-specific primers (information reg arding primers and PCR conditions used in RT-PCR will be provided upon request). All semi-quantitative cDNA fragments were amplified using TaKaRa Ex Taq^© ^(Intergen, Burlington, MA). PCR products were verified to be in the linear range and visualized by ethidium bromide staining. Images were processed using the Eagle Eye II still video system (Stratagene, La Jolla, CA). To validate the above quantitation method, PCR products also were amplified for 15 cycles (undetectable by ethidium bromide staining) and detected by standard Southern blot hybridization using their corresponding [α-^32^P]-labeled cDNA probes prepared by RT-PCR (data not shown). A minimum of three independent RT-PCR experiments were analyzed using the NIH image software program and normalized using the expression levels of GAPDH.

### Analysis of p53 and p15^INK4b ^proteins

Cell lysate protein (30 μg; determined using the Bio-Rad protein reagent) was separated by SDS-PAGE with precast 4%-20% gradient gels (Bio-Rad, Hercules, CA). Gels were electro-transferred to nitrocellulose paper, and proteins were detected with antibodies specific for p53 (SC-99, Santa Cruz Biotechnology, Santa Cruz, CA) and p15^INK4b ^(a gift from Gordon Peters), followed by horseradish peroxidase-labeled secondary antibody and either chemiluminescence or colorimetric detection systems.

### p53 functional assay

A p53-Luciferase reporter plasmid was constructed in the pGL3 Luciferase reporter vector (Promega, Madison, WI) using double-stranded oligonucleotides corresponding to tetramers of the p53 consensus binding sequence (5'-AGGCATGCCT-3') as described previously [44]. Transient transfection was carried out using electroporation (1100 μF, 275 V) as previously described [58]. For transfection, 20 μg of promoter reporter plasmids (p53-Luc) or 20 μg of control reporter plasmids (Con-Luc) [44] were cotransfected with 5 μg of pcDNA3.1-*Lac*Z plasmid. Transfection efficiency was normalized using PCR amplification of the β-lactamase (*LacZ*) gene by PCR. Gel loading was normalized using RT-PCR amplification of the GAPDH gene. Levels of luciferase activity were determined using the Luciferase Assay System (Promega) following the manufacturer's instructions.

## Authors' contributions

S.A.C. carried out isolation of the cells, cell culture, cell growth analysis, cell cycle analysis, RT-PCR analysis, p53-functional assay, conceived of the study, participated in the design and coordination of the study, and drafted the manuscript. B-W.K. performed the Western blot analysis and participated in the design and coordination of the study. M.M.L. helped perform some of the RT-PCR experiments. H.K. participated in the design and coordination of the study. D.N.F. participated in the design and coordination of the study and helped draft the manuscript. All authors read and approved the final manuscript.
